# Healthy Chilean Adolescents with HOMA-IR **≥** 2.6 Have Increased Cardiometabolic Risk: Association with Genetic, Biological, and Environmental Factors

**DOI:** 10.1155/2015/783296

**Published:** 2015-07-27

**Authors:** R. Burrows, P. Correa-Burrows, M. Reyes, E. Blanco, C. Albala, S. Gahagan

**Affiliations:** ^1^Institute of Nutrition and Food Technology, University of Chile, Avenida El Líbano 5524, Macul, 7840390 Santiago, Chile; ^2^Division of Child Development and Community Health, University of California, San Diego, 9500 Gilman Drive, MC 0927, La Jolla, San Diego, CA 92093-0927, USA

## Abstract

*Objective.* To determine the optimal cutoff of the homeostasis model assessment-insulin resistance (HOMA-IR) for diagnosis of the metabolic syndrome (MetS) in adolescents and examine whether insulin resistance (IR), determined by this method, was related to genetic, biological, and environmental factors. *Methods.* In 667 adolescents (16.8 ± 0.3 y), BMI, waist circumference, glucose, insulin, adiponectin, diet, and physical activity were measured. Fat and fat-free mass were assessed by dual-energy X-ray absorptiometry. Family history of type 2 diabetes (FHDM) was reported. We determined the optimal cutoff of HOMA-IR to diagnose MetS (IDF criteria) using ROC analysis. IR was defined as HOMA-IR values above the cutoff. We tested the influence of genetic, biological, and environmental factors on IR using logistic regression analyses. *Results.* Of the participants, 16% were obese and 9.4 % met criteria for MetS. The optimal cutoff for MetS diagnosis was a HOMA-IR value of 2.6. Based on this value, 16.3% of participants had IR. Adolescents with IR had a significantly higher prevalence of obesity, abdominal obesity, fasting hyperglycemia, and MetS compared to those who were not IR. FHDM, sarcopenia, obesity, and low adiponectin significantly increased the risk of IR. *Conclusions.* In adolescents, HOMA-IR ≥ 2.6 was associated with greater cardiometabolic risk.

## 1. Introduction

Insulin resistance (IR) is the most common metabolic alteration related to obesity and represents an important link between obesity and other metabolic and cardiovascular complications related to oxidative stress and inflammation [1]. IR is acknowledged to be critical in the development of type 2 diabetes (T2D) and has been associated with obesity, metabolic syndrome (MetS), hypertension, and ischemic cardiovascular disease [1, 2]. Although impaired beta-cell function is ultimately responsible for T2D, IR precedes beta-cell dysfunction and, thus, plays a major role in the pathogenesis of this chronic disease [2]. Insulin resistance has become a serious health issue in the pediatric and adolescent age group [3]. In children and adolescents, IR is significantly related to obesity, cardiometabolic risk, and inflammation [3–6]. Family history of type 2 diabetes (FHDM), ethnicity, pre- and postnatal nutritional environment, obesity, puberty, diet, and sedentary lifestyle can all influence insulin sensitivity in the pediatric population [4].

In many developing countries, the nutritional transition and particularly the “westernization” of life-styles have caused a significant rise in obesity and comorbidities associated with IR, including T2D and ischemic cardiovascular disease [7–9]. Chile is a middle income country that underwent a profound shift from under- to overnutrition in less than two decades. Western dietary patterns and inactive lifestyles are widely spread in all age groups, especially among people from middle-low and low to middle socioeconomic status (SES) [10, 11]. The prevalence of obesity in Chilean children and adolescents more than tripled (5% to 17%), since the early 1990s [12, 13]. In a study of obese children and adolescents, 53% and 30% had IR and MetS, respectively [14]. According to evidence from another sample of obese Chilean adolescents, insulin resistance was associated with higher risk of Mets [15].

Although hyperinsulinemic–euglycemic clamp is the gold standard method for assessment of insulin sensitivity, it is expensive and invasive. Alternative methods based on surrogate markers derived from fasting insulin and glucose, such as the homeostasis model assessment-insulin resistance (HOMA-IR), have been validated and proposed [16, 17]. HOMA-IR values ≥ 2.5 indicate IR in adults [17], but the corresponding cutoff value for children and adolescents has not been determined [4]. In many studies, IR diagnosis is based on HOMA-IR distribution in a reference population [15, 18]. MetS in pediatric population has been considered for defining the HOMA-IR cutoff point for IR diagnosis, in several contexts around the world [19, 20].

This research aims to determine the optimal cutoff value of HOMA-IR for MetS diagnosis in healthy adolescents, to examine whether IR assessed by using this cutoff value is related to anthropometric, metabolic, and cardiovascular risk profile, and to evaluate the association of IR with genetic, biological, and environmental factors.

## 2. Methods and Procedures

### 2.1. Study Design and Population

We studied 667 16- to 17-year-old adolescents living in urban Santiago, from low to middle SES neighborhoods, who were part of an iron deficiency anemia preventive trial and follow-up study beginning in infancy [21]. The participants were assessed in adolescence to understand biological and psychosocial determinants of adolescent obesity and cardiovascular risk. The study was approved by the institutional review boards of the University of Michigan, Institute of Nutrition and Food Technology (University of Chile), and the University of California San Diego. Participants and their primary caregiver provided informed and written consent, which was obtained according to the norms for Human Experimentation, Code of Ethics of the World Medical Association (Declaration of Helsinki, 1995).

### 2.2. Measurements

#### 2.2.1. Anthropometry and Body Composition

A research physician used standardized procedures to measure the adolescent's height (cm) to the nearest 0.1 cm, using a Holtain stadiometer and weight (kg) to the nearest 0.1 kg using a Seca scale. Body mass index (BMI) (Kg/m^2^) was calculated and obesity status was calculated according to WHO references. Measurements were taken twice, with a third measurement, if the difference between the first two exceeded 0.3 Kg for weight and 0.5 cm for height. Waist circumference was measured with nonelastic flexible tape at the high point of the iliac crest around the abdomen and recorded to 0.1 cm. Measurements were taken twice, with a third measurement, if the difference between the first two exceeded 1.0 cms. Dual-energy X-ray absorptiometry (DEXA) was used to measure fat mass (%) and fat-free mass (%). Fat-Free Mass Index (FFMI) was estimated according to Wells and Fewtrell [22]. FFMI values were expressed as percentage of BMI; values ≤ 25th percentile in our sample were considered sarcopenia, after adjusting for sex.

#### 2.2.2. Additional Cardiovascular Risk Markers

After 15 minutes at rest and prior to the physical examination, systolic and diastolic blood pressures (SBP and DBP) were measured, three times on the nondominant arm using a standard mercury sphygmomanometer; the average value was used for analyses. Fasting serum total glucose (Gli), cholesterol, triglycerides (TG), high-density lipoprotein (HDL), insulin, adiponectin, and high sensitivity C-reactive protein (hs-CRP) levels were performed after a 12-hour overnight fast. Radioimmunoassay (RIA DCP Diagnostic Products Corporation, LA, USA) was used for insulin and adiponectin determinations. High sensitivity C-reactive protein (hs-CRP) was measured with a sensitive latex-based immunoassay (latex-enhanced nephelometry method). Glucose was measured with enzymatic-colorimetric test (QCA S.A. Amposta, Spain) and cholesterol profile (Col-HDL and TG mg/dL) was determined by analytical methodology dry (Vitros, Johnson & Johnson, Clinical Diagnostics Inc.). Values of hs-CRP ≥ 1.1 mg/L (75th percentile in our sample) were considered low-grade systemic inflammation and adiponectin ≤ 7.9 *μ*g/mL (25th percentile in our sample) was considered to be low. HOMA-IR was calculated and the optimal cutoff value of HOMA-IR to diagnose MetS was determined with receiver operating characteristic (ROC) regression methodology [23] and used for the diagnosis of IR. CVRF and MetS were diagnosed based on the 2007 International Diabetes Federation (IDF) consensus statement on the clinical definition of the MetS in the pediatric age range. These criteria include central obesity plus 2 of the 4 following factors: abdominal obesity (WC ≥ 80 and 90 cm in females and males, resp.), high blood pressure (SBP ≥ 130, DBP ≥ 85), hypertriglyceridemia (TG ≥ 150 mg/dL), low HDL (≤50 and ≤40 mg/dL in female and male adolescents, resp.) and fasting hyperglycemia (Gli ≥ 100 mg/dL) [24].

#### 2.2.3. Diet and Physical Activity

Food intake and physical activity (PA) habits were evaluated using validated and standardized self-report questionnaires, scored from 0 to 10, with higher scores denoting more nutritious food intake or more physical activity [25, 26]. The questionnaires were administered by a researcher during the half-day assessment. The quality of food intake was measured by the amount of saturated fat, fiber, sugars, and salt in the food items. We applied cutoffs established by Burrows et al. [25] to classify the eating habits of participants into three groups: unhealthy (scores ≤ 25th percentile), intermediate (scores 26th to 74th percentile), and healthy (scores ≥ 75th percentile). PA was measured by the total amount of time devoted to sedentary activities, recreational games, active commuting, and weekly scheduled exercise either school or nonschool organized. Participants with scores ≤ 25th percentile were considered as physically inactive, those with scores ≥ 75th percentile were physically active, and those in between were moderately active.

#### 2.2.4. Family History of Type 2 Diabetes

A standardized questionnaire of FHDM, including first- and second-degree relatives, was answered by the participant's primary caregiver (father, mother, or grandparents). Reporting FHDM in at least one parent or grandparent was required to fit our definition of positive FHDM.

### 2.3. Statistical Analysis

Data were analyzed using Stata for Windows version 12.0 (Lakeway Drive College Station, TX, US). A *P* value of <0.05 denoted statistical significance. Statistical analysis included chi-square tests for categorical variables and Student's *t*-test for comparison of mean values of anthropometric and cardiometabolic variables. ROC analysis was used to find the optimal cutoff of HOMA-IR for diagnosis of IR. Bivariate analyses were used to test the association between obesity, sarcopenia, unhealthy food intake, physical inactivity, and FHDM and the outcome, IR. We used multiple logistic regressions to assess the influence of the variables significantly associated with IR. We excluded data on participants who reported unknown information in at least one parent or grandparent (*n* = 125). Four models were estimated. The first one included FHDM and low PA as independent variables. In the second model, obesity was added. The third model included sarcopenia. Finally, a full adjusted model contained all mentioned covariates with the addition of low adiponectin. The likelihood ratio test was performed to test the overall significance of all coefficients in the multiple adjusted models, whereas the Hosmer-Lemeshow goodness-of-fit test was used to evaluate their effectiveness in predicting the outcome of IR.

## 3. Results and Discussion

### 3.1. Results

#### 3.1.1. Patients' Characteristics

Our sample was made up of 52.2% male and 47.8% female adolescents who were on average 16.8 (SD 0.3) years old. The prevalence of obesity was 16.2% whereas 9.4% of participants met criteria for MetS.

#### 3.1.2. Optimal Cutoff Point for the Diagnosis of Insulin Resistance

**Figure 1:**
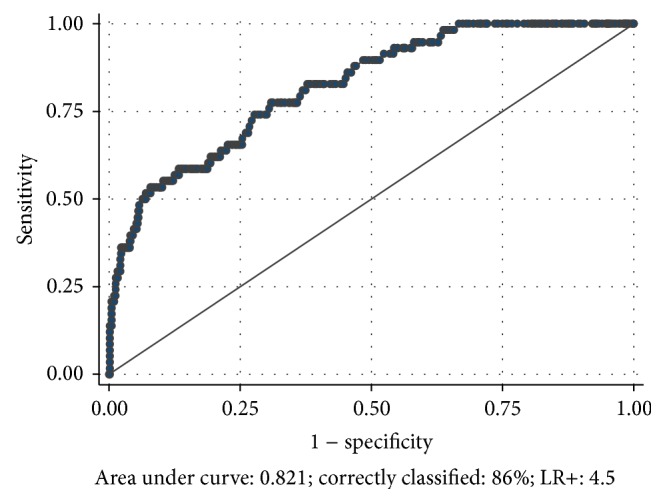
ROC curve to determine the optimal cutoff value of HOMA-IR for MetS diagnosis in healthy adolescents.

A HOMA-IR value of 2.6 showed the best sensitivity (59%) and specificity (87%) for diagnosing MetS (AUC: 0.821; correctly classified: 87%; LR+: 4.5) (Figure 1). According to this optimal cutoff point, 16.3% of adolescents in the sample had IR.

**Figure 2:**
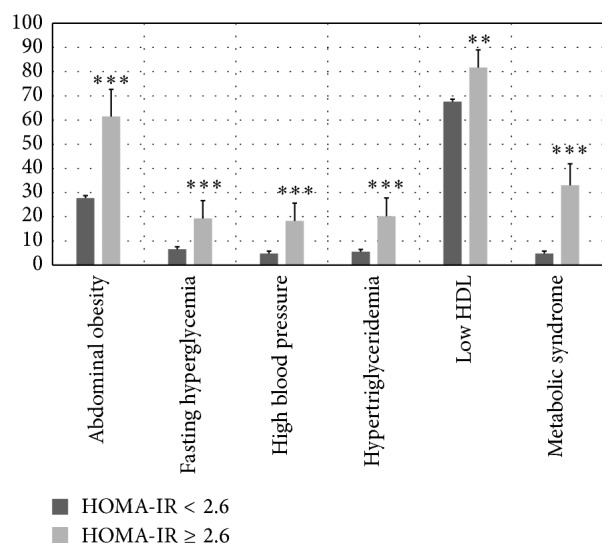
Prevalence rates of cardiovascular and metabolic risk factors by HOMA-IR. Error bars are 95% CI. Statistical significant difference by Pearson's Chi2. Significance level: ^*^ *P* < 0.05; ^**^ *P* < 0.01; ^***^ *P*<0.001.

As shown in Table 1, adolescents with IR (HOMA-IR ≥ 2.6) had significantly higher (*P* < 0.001) BMI *z* score, WC, fat mass (%), systolic and diastolic BAP, total cholesterol, LDL-chol, triglyceride, glucose, and insulin, as well as significantly lower mean values of lean mass (%), HDL-chol, and adiponectin compared to adolescents with HOMA-IR < 2.6. Obesity (40.4% versus 9.3%; *P* < 0.001) and physical inactivity (51.4% versus 37.4; *P* = 0.006) were significantly higher among participants with IR compared to the insulin sensitive group. Similarly, adolescents with IR (Figure 2) show a significantly higher prevalence of abdominal obesity (61.5% versus 27.8%; *P* < 0.001), high blood pressure (18.4% versus 4.8%; *P* < 0.001), fasting hyperglycemia (19.3% versus 6.6%; *P* < 0.001), hypertriglyceridemia (20.4% versus 5.6%; *P* < 0.001), and MetS (33.0% versus 4.8%; *P* < 0.001).

#### 3.1.3. Association of IR with Genetic, Biological, and Environmental Factors

Table 2 contains the results of the bivariate analysis that was used to identify explanatory variables of IR in adolescents. We found a significant association between IR and obesity (OR: 6.6; 95% CI: 4.1–10.6), sarcopenia (OR: 4.9; 95% CI: 3.2–7.5), low adiponectin (OR: 2.5; 95% CI: 1.6–4.0), physical inactivity (OR: 1.8; 95% CI: 1.2–2.7), and FHDM (OR: 1.7; 95% CI: 1.1–2.9).

Table 3 shows the results of the multivariate logistic regression analyses assessing the relationship between genetic, biological, and environmental variables significantly associated in the bivariate analyses with the outcome, IR. Those with physical inactivity had significantly higher odds of IR in Model 1. However, the association was lost when sarcopenia, obesity, and low adiponectin were entered in the regression equation. In a fully adjusted model, FHDM (OR: 1.79; 95% CI: 1.1–3.1), sarcopenia (OR: 1.9; 95% CI: 1.1–3.6), obesity (OR: 2.4; 95% CI: 1.2–4.9), and low adiponectin (OR: 2.3; 95% CI: 1.3–3.8) significantly increased the risk of IR.

### 3.2. Discussion

In this cohort of healthy Chilean adolescents of low to middle SES, the optimal cutoff point of HOMA-IR for IDF MetS diagnosis was 2.6 (sensitivity: 59% and specificity: 87%). Adolescents with HOMA-IR ≥ 2.6 showed a significantly higher prevalence of obesity (40.4%), abdominal obesity (61.5%), hypertriglyceridemia (20.4%), high blood pressure (18.4%), fasting hyperglycemia (19.3%), and MetS (33.0%). Thus, HOMA-IR of 2.6 or higher would find young individuals with greater biological risk.

In adolescents, HOMA-IR values ranging from 2.2 to 5.3 have been reported as cutoff for diagnosing MetS, but these studies vary greatly in their design, sample size, age and nutritional status of participants, and degree of pubertal development [14, 15, 19, 20, 27–29]. Our findings are similar to population-based studies in a number of other contexts [20, 28]. In Chinese children aged 6 to 18 years, 2.6 was the optimal cutoff point of HOMA-IR for MetS ATP III diagnosis [20]. In urban adolescents from India, aged 10 to 18 years, HOMA-IR value of 2.5 had the optimal sensitivity (>70%) and specificity (>60%) for MetS ATP III and IDF diagnosis [28]. This cutoff was most likely to detect MetS in adolescents from different BMI categories [28]. Other clinical studies also agree with our finding. In prepubertal overweight Brazilian children, HOMA-IR value of 2.5 showed optimal sensitivity (61%) and specificity (74%) for MetS diagnosis [30].

In this sample, FHDM, obesity, sarcopenia, and low adiponectin were all independently associated with a significant increased risk of IR. All these factors have been recognized as important determinants of IR and TD2 in pediatric populations [3, 4, 31–37]. In studies of twins, 50% of the variance in insulin sensitivity and secretion was attributed to genetic factors [31]. Furthermore, healthy children with FHDM were more likely than those without to be insulin resistant, with an impaired balance between insulin sensitivity and secretion [32]. Moreover, obesity is the most prevalent pathophysiological determinant of IR, whereas insulin sensitivity is inversely associated with BMI and percent body fat in child and adolescent populations [33]. Similarly, muscular strength has been identified as an independent and powerful predictor of better insulin sensitivity and, conversely, lower muscular strength and higher central adiposity are highly predictive of higher levels of IR in healthy children and adolescent [34]. Moreover, there is growing evidence that exercise exerts beneficial effects partly through alterations in the adipokine profile; that is, exercise increases secretion of anti-inflammatory adipokines, improves metabolic syndrome and insulin sensitivity, and reduces proinflammatory cytokines [35]. Finally, in children, adiponectin levels are lowered with MetS and obesity and inversely related to IR [36]. In both liver and skeletal muscle, adiponectin reduces triglyceride content and improves insulin signaling by increasing the gene expression involved in fatty acid oxidation and decreases the hepatic glucose production by inhibiting the expression of hepatic gluconeogenic enzymes [37]. Although IR has been shown to be linked with a chronic inflammatory state, we did not find significantly lower levels of hs_CRP in adolescents with HOMA-IR < 2.6. However, it is important to emphasize that the observed levels of hs_CRP in this sample overall were higher than those reported by others using adolescent participants as well as the same measurement methodology [38, 39]. Psychological factors related to stress, a diet high in saturated fat and refined sugars, and physical inactivity may be determinants of a systemic chronic inflammatory state [40–42]. These factors are usually more prevalent among low-SES groups [43, 44], and they all have been observed in this cohort. Whereas only 7.5% (95% CI: 5.5–9.5) of participants had healthy dietary habits, 19.3% (95% CI: 16.3–22.3) were considered to be physically active (data not shown), according to national references [16]. Similarly, results from a previous work suggest that adolescents, in our cohort, are exposed to a stressful environment [45].

This research has limitations that should be considered when interpreting its results. One limitation is that we estimated sarcopenia from DEXA scans and calculated fat-free mass rather than a more direct method of assessing muscle mass [46]. Nonetheless, this methodology has better sensitivity for estimating the ratio between muscle and fat tissue compared to BMI [22, 47]. Another limitation is that our sample is not representative of the Chilean adolescent population. Our sample is composed of adolescents from low to middle SES living in urban Santiago. However, our findings may be equally relevant for a number of reasons. According to the Chilean National Health Survey, the prevalence of obesity, TD2, and CVD is significantly higher among low to middle SES individuals [48]. On the other hand, low to middle SES Chilean adolescents are highly exposed to insulin resistance risk factors including obesity, physical inactivity, low fitness, and processed food full of fat and sugar [10, 12, 25, 49–52]. Our study also makes an important contribution in confirming that obesity, sarcopenia, and FHDM might be considered relevant risk factors for IR in adolescents.

## 4. Conclusions

This research provides results that confirm that in adolescents a value of HOMA-IR ≥ 2.6 is associated with greater cardiovascular and metabolic risk [20, 28, 30]. We recommend this cutoff for diagnosis of IR in clinical practice. Adolescents with IR show a significantly higher prevalence of obesity, abdominal obesity, fasting hyperglycemia, and HFDM. In Chilean adult populations, fasting hyperglycemia, obesity, and self-reported FHDM were the best predictors of T2DM [53]. Obesity, sarcopenia, and FHDM might be considered relevant risk factors for insulin resistance. Our findings highlight the need for robust public policies and programs to reduce the risk for obesity and associated conditions in youth. For families with a history of diabetes mellitus, pediatricians should strongly counsel the youths and their parents to engage in healthy nutritional and physical activity practices in order to prevent the onset of early cardiometabolic risk.

## Figures and Tables

**Table 1:** Background characteristics of adolescents in the sample by insulin sensitivity (*n* = 667).

	HOMA-IR < 2.6 (*n* = 558)	HOMA-IR ≥ 2.6 (*n* = 109)	Total sample	*P* value^c^
Age (years)	16.8 ± 0.3^a^	16.9 ± 0.2	16.8 ± 0.3	n.s.
Anthropometrics				
BMI (*z* score)	0.48 ± 1.1	1.53 ± 1.2	0.65 ± 1.2	<0.001
WC (cm)	79.4 ± 9.9	90.4 ± 14.3	81.2 ± 11.4	<0.001
Lean Mass (%)	68.7 ± 11.4	62.0 ± 9.4	67.6 ± 11.1	<0.001
Fat mass (%)	27.9 ± 10.7	34.7 ± 9.4	29.0 ± 10.8	<0.001
BMI *z* ≥ 2 SD	52 (9.3)^b^	44 (40.4)	96 (14.4)	<0.001^d^
CVM profile				
SBP (mm Hg)	111.1 ± 10.2	118.3 ± 10.7	112.2 ± 10.6	<0.001
DBP (mm Hg)	68.7 ± 7.0	72.1 ± 6.5	69.3 ± 7.0	<0.001
TG (mg/dL)	82.5 ± 45.5	118.2 ± 71.6	88.3 ± 50.1	<0.001
HDL-chol (mg/dL)	40.8 ± 10.6	36.6 ± 10.2	40.1 ± 10.6	<0.001
Glucose (mg/dL)	87.4 ± 8.8	94.2 ± 10.8	88.6 ± 9.5	<0.001
Insulin (*µ*IU/dL)	6.3 ± 2.5	17.5 ± 7.0	8.1 ± 5.5	<0.001
hs-CRP (mg/L)	0.95 ± 1.9	1.10 ± 1.6	0.98 ± 1.9	n.s.
Adiponectin (*µ*g/mL)	12.3 ± 5.5	9.2 ± 5.1	11.8 ± 5.5	<0.001
Lifestyles habits				
Unhealthy eating	139 (24.9)	20 (18.4)	159 (23.8)	n.s.
Physical inactivity	209 (37.4)	56 (51.4)	265 (39.7)	0.006^d^

^a^Mean ± S.D; ^b^ *n* (%); ^c^Student's *t*-test, except as indicated. ^d^Chi2 (Pearson).

**Table 2:** Association between IR and sex, obesity, sarcopenia, low adiponectin, life-style habits, and HFDM2 in adolescents (*n* = 667).

	Subjects with insulin resistance (HOMA-IR ≥ 2.6)		Crude OR^a^
	*n*	%	
Overall	109	16.32	
Obesity			
No	65	11.4	Ref. group
Yes	44	45.8	6.60 [4.09–10.6]^***^
Sarcopenia			
No	50	10.8	Ref. group
Yes	59	35.1	4.87 [3.17–7.50]^***^
Low adiponectin			
No	74	13.6	Ref. group
Yes	35	28.5	2.53 [1.59–4.01]^***^
Physical inactivity			
No	53	13.2	Ref. group
Yes	56	21.1	1.77 [1.17–2.67]^***^
Unhealthy food intake			
No	89	17.5	Ref. group
Yes	20	12.6	0.68 [0.40–1.14]^***^
Family history T2D^b^			
No	23	11.7	Ref. group
Yes	74	18.8	1.73 [1.10–2.88]^***^

^a^Statistical significance: ^*^ *P* < 0.05, ^**^ *P* < 0.01, and ^***^ *P* < 0.001; obesity: BMI *z*-score ≥2 SD; sarcopenia: % FFMI ≤25th percentile; low adiponectin: adiponectin ≤7.9 *μ*g/mL; physical inactivity: PA score ≤3; unhealthy food intake: eating test score ≤4.4; FHDM^b^:family history of type 2 diabetes in at least one first-degree relative (*n* = 590).

**Table 3:** Influence of FHDM, physical inactivity, sarcopenia, obesity, and low adiponectin over the risk of insulin resistance (*n* = 590).

	Model 1	Model 2	Model 3	Model 4
	OR [95% CI]	OR [95% CI]	OR [95% CI]	OR [95% CI]
FHDM	1.74 [1.10–2.88]^*^	1.74 [1.10–2.94]^*^	1.72 [1.10–2.92]^*^	1.80 [1.10–3.08]^*^
Physical inactivity	1.77 [1.14–2.75]^*^	1.49 [0.94–2.37]	1.42 [0.89–2.26]	1.45 [0.90–2.34]
Sarcopenia	(⋯)	4.25 [2.67–6.75]^***^	2.27 [1.25–4.12]^**^	2.56 [1.21–4.07]^**^
Obesity	(⋯)	(⋯)	3.25 [1.71–6.19]^***^	2.92 [1.51–5.66]^**^
Low adiponectin	(⋯)	(⋯)	(⋯)	2.22 [1.30–3.77]^**^

Likelihood ratio (Chi2)	11.46^**^	48.5^***^	61.6^***^	69.8^***^
Hosmer-Lemeshow	<0.001 (0.99)	0.39 (0.99)	0.18 (0.99)	1.21 (0.98)
Correctly classified	83.6%	83.6%	84.2%	84.6%

OR [95% CI]: odd ratio [95% confidence interval]. Statistical significance: ^*^ *P* < 0.05; ^**^ *P* < 0.01; ^***^ *P* < 0.001. [⋯]: nonobserved. OR [95% CI]: odd ratio [95% confidence interval]. FHDM: family history of type 2 diabetes in at least one first-degree relative. Obesity: BMI *z* score ≥2 SD; sarcopenia: FFMI ≤25th percentile; physical inactivity: PA score ≤3; low adiponectin: adiponectin ≤7.9 *μ*g/mL.
